# Hadamard Transform Ion Mobility Spectrometry Based on Matrix Encoding Modulation

**DOI:** 10.3390/s23146267

**Published:** 2023-07-10

**Authors:** Ke Chen, Lingfeng Li, Peng Li

**Affiliations:** School of Electronic and Information Engineering, Soochow University, Suzhou 215006, China; kchenchenke@stu.suda.edu.cn

**Keywords:** IMS, Hadamard transform, SNR, matrix

## Abstract

Ion mobility spectrometry (IMS) has been widely used for the on-site detection of trace chemicals, but continue to suffer from a low duty cycle of ion injection. The Hadamard transform ion mobility spectrometry (HT-IMS) technique was employed to address the problem with increased signal-to-noise ratio (SNR). However, in this work, through simulation, a certain deviation between the mathematical principle of Hadamard transform and actual data collection process was found, which resulted in a distortion of the baseline in the spectrum. The reason behind this problem was analyzed and a novel IMS based on Sylvester-type Hadamard matrix encoding modulation (Sylvester-HT-IMS), together with a set of date collection and processing technique, was proposed. Sylvester-HT-IMS offered much improved quality of deconvoluted spectrum and overall performance in the simulation. In experimental verification, with reactant ions and product ions characterized, Sylvester-HT-IMS showed improved SNR and ion discrimination over both conventional signal-averaged IMS (SA-IMS) and HT-IMS, providing an alternative method for multiplexed IMS.

## 1. Introduction

Drift time ion mobility spectrometry (DT-IMS) is a trace compound analysis and detection technique, which is widely used in the detection of explosives [1,2,3], drugs [4,5] and chemical warfare agents [6,7] due to its ambient pressure working condition, low detection limit, short measurement time, compact size and easy operation.

However, one of the major drawbacks of DT-IMS is its inherent low utilization rate of analytes. In order to maintain the necessary resolving power (Rp), during a traditional DT-IMS measurement, the ion gate pulses once for a span of a few hundred microseconds before the ion gate closes again. When comparing the opening time of the ion gate (tens of microseconds) to the duration of the ion mobility measurement (tens of milliseconds), the duty cycle of the DT-IMS is about 1%. This low duty cycle causes significant analyte loss and extremely low ion utilization efficiency in cases of trace detection where the amount of sample may be limited, particularly given that when the ion gate is closed, the reverse electric field which prevents ions from entering the drift area will form a depletion region [8] where all ions will be discharged. It is difficult for certain ions, especially slower ions, to pass through the depletion region and be detected as a result of the low duty cycle, causing poor SNR for slower ions and the so-called ion discrimination effect [9]. Unfortunately, although simply increasing the duty cycle can improve these issues, it worsens the Rp of the spectrum, leading to extra efforts in data processing and analysis for spectrum [10].

To address these issues, Clowers and Szumlas [11,12] introduced HT-IMS in the same year, which employs ion gate modulation based on a pseudo-random binary sequence (PRBS) [13] and increases the duty cycle of IMS to 50%. The Hadamard multiplexing technique significantly improves the SNR of DT-IMS compared to the average method [11]. Subsequently, researchers have proposed numerous novel approaches to eliminate the defects of HT-IMS to further improve its performance, including inverting the modulation sequence [14,15,16], spectral reconstruction [17], continuously varying the duty cycle of the modulated pulse [18], modifications to the modulation sequence [19,20,21], the application of false peak detecting and minimizing algorithms [22,23]. These works focused on the improvement of HT-IMS using various measurement methods and data processing techniques; however, they failed to discover the deviation between the mathematical description of Hadamard transform and the actual signal acquisition process of IMS.

In this work, we conducted an examination of the underlying mathematical principle of the Hadamard transform method and its correlation with the IMS data collecting process. Subsequently, a novel multi-pulse IMS called Sylvester-HT-IMS was developed to implement improvements in the signal acquisition and processing of HT-IMS. Finally, SA-IMS, HT-IMS and Sylvester-HT-IMS were characterized for both the SNR and ion discrimination effect, respectively, through two rigorous experiments on reactant ions and product ions.

## 2. Principle and Applicability of HT-IMS

In HT-IMS, PRBS a was utilized to modulate IMS, and the detected signal on the detector was the superimposition of multiple spectra with different starting times as follows [11,12]:

(1) $$S_{n}X=Y$$

Among them, ***X*** represents the desired spectrum, Y denotes the superimposed spectrum detected by the detection plate and $S_{n}$ is the *n*-order matrix constructed by PRBS. The specific construction method involves using the PRBS a as the first column of $S_{n}$ and then performing circular shifting *i* times on a to construct the (*i + 1*)-th column of $S_{n}$. Therefore, the deconvolution of HT-IMS can be expressed by the following Equation [11,12].

(2) $$X=S_{n}^{-1}Y$$

We assume that PRBS is *s*_1_, *s*_2_, *s*_3_, *s*_4_, *s*_5_, *s*_6_, *s*_7_ (1101001), where each bit has a duration of *d*. Using the aforementioned method, $S_{7}$ is constructed assuming that the sampling point of the superimposed signal is *A B C D E F G*, and the corresponding desired spectrum data point is *a b c d e f g* with a total spectrum time window of 6*d*. The collection process can be expressed in the following equation according to Equation (1) [24]:

(3) $$\;\left[\begin{array}{c} A\\ B\\ C\\ D\\ E\\ F\\ G \end{array}\right]=\left[\begin{array}{ccccccc} s_{1} & s_{7} & s_{6} & s_{5} & s_{4} & s_{3} & s_{2}\\ s_{2} & s_{1} & s_{7} & s_{6} & s_{5} & s_{4} & s_{3}\\ s_{3} & s_{2} & s_{1} & s_{7} & s_{6} & s_{5} & s_{4}\\ s_{4} & s_{3} & s_{2} & s_{1} & s_{7} & s_{6} & s_{5}\\ s_{5} & s_{4} & s_{3} & s_{2} & s_{1} & s_{7} & s_{6}\\ s_{6} & s_{5} & s_{4} & s_{3} & s_{2} & s_{1} & s_{7}\\ s_{7} & s_{6} & s_{5} & s_{4} & s_{3} & s_{2} & s_{1} \end{array}\right]\left[\begin{array}{c} a\\ b\\ c\\ d\\ e\\ f\\ g \end{array}\right]$$

Namely:

(4) $$\left\{\begin{array}{c} A=s_{1}a+s_{7}b+s_{6}c+s_{5}d+s_{4}e+s_{3}f+s_{2}g\\ B=s_{2}a+s_{1}b+s_{7}c+s_{6}d+s_{5}e+s_{4}f+s_{3}g\\ \ldots \\ G=s_{7}a+s_{6}b+s_{5}c+s_{4}d+s_{3}e+s_{2}f+s_{1}g \end{array}\right.$$

**Figure 1:**
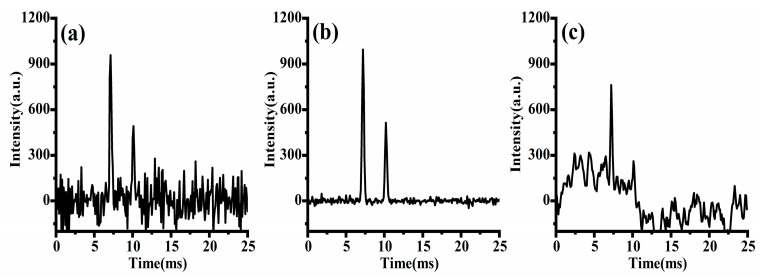
Simulated spectrum of (**a**) single pulse; (**b**) ideal Hadamard transform; (**c**) actual Hadamard transform.

**Figure 2:**
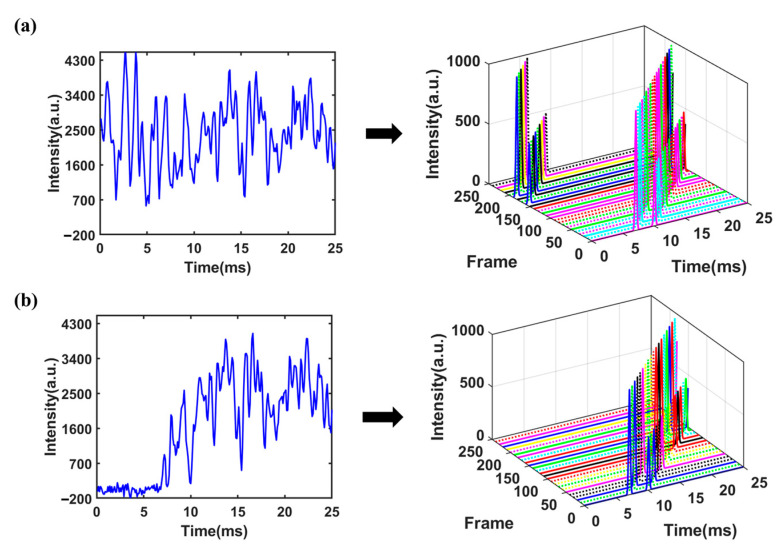
Decomposition diagram of multi-pulse ion implantation: (**a**) mathematically ideal situation; (**b**) actual data collection.

From Equation (4), it is evident that the sampling point *A* of the superimposed signal at the starting time is a linear combination of the sampling points *a b c d e f g* in the single-pulse spectrum, with combination coefficients of either 0 or 1. This implies that point *A* at the starting time in the superimposed signal would contain information about all the sampling points of the single-pulse spectrum from time 0 to 6*d*. MATLAB (MathWorks, Natick, MA, USA) was used to simulate the above unreachable signal acquisition process and make a comparison with the actual situation. The simulated spectrum depicted in Figure 1a contains two ion peaks fitted with Gaussian function, with peak heights of 1000 and 500, respectively. The peaks were located at 6.5 ms and 10.5 ms, respectively. Gaussian white noise with a level of 40 dB was added to the spectrum. The ion gate was modulated using a PRBS with a length of 255 and pulse width of 100 μs. The obtained superimposed signal was decomposed into 255 frame single-pulse spectra as shown in Figure 2a (for clarity, no noise has been added to the single-pulse spectrum and different frames are marked with different colors), and the spectrum obtained by binary number 1 was marked with solid lines, while the spectrum obtained by binary number 0 was marked with dashed lines. It is worth noting that under ideal circumstances, the portion of a single-pulse spectrum exceeding 25.5 ms in each frame needs to be cyclically shifted to the starting time of the spectrum. The desired spectrum obtained through deconvolution is shown in Figure 1b. It can be seen that the peak-to-peak value of noise has a significant reduction compared to that of the single-pulse spectrum.

However, considering the actual IMS measurements where the signal generated by an ion swarm at the detector is always delayed from the injection through the ion gate, when the time window of ion gate modulation and data collection is aligned as required by deconvolution, the spectrum or ion signal will be mismatched from its corresponding ion injection, as shown in Figure 2b. Signals from ion injections in the later part of the sequence are not included in the convoluted spectrum and therefore will lead to distortion of the deconvoluted spectrum, as shown in Figure 1c. The origin of this commonly observed distortion of the deconvoluted spectrum of HT-IMS is the deviation of the mathematical model and actual data acquisition process, rather than imperfect measurements.

Given that a spectrum is a two-dimensional signal that varies with time, it is necessary to establish a new mathematical model for multi-pulse IMS. The measurement process of multi-pulse IMS can be represented as follows:

(5) $$\varphi_{i}=w_{i1}f_{1}+w_{i2}f_{2}\ldots +w_{ii}f_{i}\ldots +w_{in}f_{n}+e_{i}\left(i=1,2,\ldots ,n\right)$$

Namely:

(6) $$\varphi_{i}=\left(f_{1},f_{2}\ldots f_{i}\ldots f_{n}\right){w_{i}}^{T}+e_{i}$$

where $\varphi_{i}$ is the superimposed signal obtained by modulating the ion gate through a binary sequence; the value of $w_{ij}$ is either 1 or 0, indicating whether the ion gate is open or closed. $f_{i}$ represents the single-pulse spectrum which consists of *n* sampling points and is obtained from the *i*-th pulse, while $e_{i}$ represents the measurement error associated with the combined measurement. It is important to note that $e_{iq}$ is independent of $f_{iq}$ and meets the mathematical expectation $E\left\{e_{iq}\right\}=0$, $E\left\{e_{iq}e_{ip}\right\}=\sigma^{2}\delta \left(p,q\right)$, where $\sigma^{2}$ represents the variance of the measurement error.

(7) $$\delta \left(i,j\right)=\left\{\begin{array}{c} 1\;i=j\\ 0\;i\neq j \end{array}\right.$$

In order to extract $f_{i}$ from the superimposed signal, a total of n combined measurements are required and n corresponds to the number of sampling points contained in $f_{i}$. Let the coefficient matrix of n measurements be W. Let the error matrix of combined measurement be $E=\left(e_{1},e_{2},\ldots e_{n}\right)$, written in matrix form:

(8) φ=fW+E 

Assuming that the coefficient matrix W is invertible, and the inverse matrix is $W^{-1}=\left(\gamma_{ij}\right)$, according to Equation (5), the unbiased estimate of f is $\hat{f}$:

(9) $$\hat{f}=\varphi W^{-1}=f+EW^{-1}$$

Then, the mean square error of $f_{i}$ is:

(10) $$\xi_{i}=\frac{1}{n}\sum_{j=1}^{n}{\left({\hat{f}}_{ij}-f_{ij}\right)}^{2}=\sigma^{2\;}({\gamma_{i1}}^{2}+{\gamma_{i2}}^{2}+\ldots +{\gamma_{in}}^{2})\;$$

namely:

(11) $$\;\xi =\frac{\sigma^{2}}{n}\sum_{i=1}^{n}\sum_{j=1}^{n}{\left(\gamma_{ij}\;\right)}^{2}=\frac{\sigma^{2}}{n}tr{\left(W^{T}W\right)}^{-1}$$

The SNR of combined measurement is:

(12) $$SNR=\frac{f}{\sqrt{\frac{\sigma^{2}}{n}tr{\left(W^{T}W\right)}^{-1}}}=\frac{\sqrt{n}f}{\sqrt{tr{\left(W^{T}W\right)}^{-1}}\sigma }$$

The SNR of the single-pulse spectrum is:

(13) $$\;SNR=\frac{f}{\sigma }$$

The gain of SNR of every spectrum in $\left(f_{1},f_{2}\ldots f_{i}\ldots f_{n}\right)$ relative to the single-pulse spectrum is [25]:

(14) $$G=\sqrt{\frac{n}{tr{\left(W^{T}W\right)}^{-1}}}$$

A Sylvester-type Hadamard matrix [26] is employed to construct the W matrix. The order of the Sylvester-type Hadamard matrix is a power of 2, and it can be recursively constructed from low order to high order. The construction process is as follows:

(15) $$H_{2n}=\left[\begin{array}{cc} H_{n} & H_{n}\\ H_{n} & -H_{n} \end{array}\right]\;\left(n=2^{m},m=1,2,3\ldots \right)$$

where the 2-order Hadamard matrix is

(16) $$H_{2}=\left[\begin{array}{cc} 1 & 1\\ 1 & -1 \end{array}\right]$$

**Figure 3:**
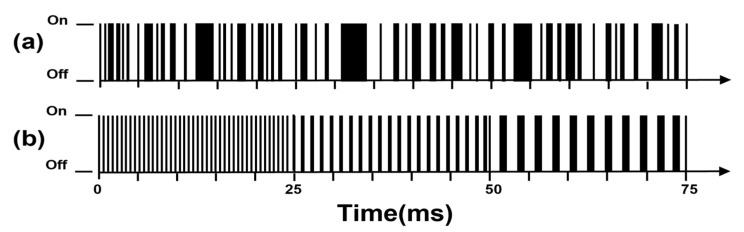
The ion gate pulse: (**a**) HT-IMS; (**b**) Sylvester-HT-IMS.

By excluding the first row and first column of the *n*-order Sylvester-type Hadamard matrix $H_{n}$ and replacing all -1 elements in the resulting square matrix with 0 elements, $H_{n-1}$ can be obtained. Then, the IMS is modulated with each row element of matrix $H_{n-1}$. The comparison between the pulse sequences of Sylvester-HT-IMS and HT-IMS is shown in Figure 3. The uniformity of the distribution of binary numbers 0 and 1 is enhanced compared to PRBS used in traditional HT-IMS.

$H_{n-1}$ meets:

(17) $$H_{n-1}^{-1}=\frac{2}{n}\left(2H_{n-1}^{T}-J_{n-1}\right)=\frac{2}{n}H_{T}$$

where $H_{n-1}^{T}$ is the transposition of $H_{n-1}$, $J_{n-1}$ is the full 1 matrix and $H_{T}$ is the new matrix obtained by replacing the 0 in $H_{n-1}$ with −1. Thus, it is evident that through a straightforward numerical substitution step, the inverse matrix of $H_{n-1}$ can be quickly computed, thereby reducing the computational complexity of deconvolution. It can be inferred from the above equation that:

(18) $$tr{\left({H_{n-1}}^{T}H_{n-1}\right)}^{-1}=\frac{{4(n-1)}^{2}}{n^{2}}\approx 4$$

(19) $$G=\sqrt{\frac{n-1}{tr{\left({H_{n-1}}^{T}H_{n-1}\right)}^{-1}}}\approx \frac{\sqrt{n-1}}{2}$$

**Figure 4:**
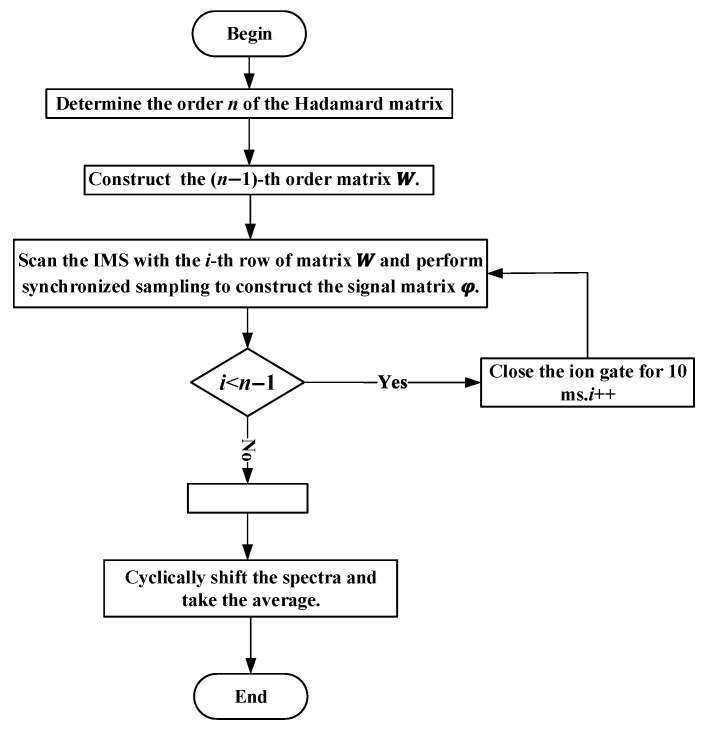
Signal modulation and processing flowchart.

**Figure 5:**
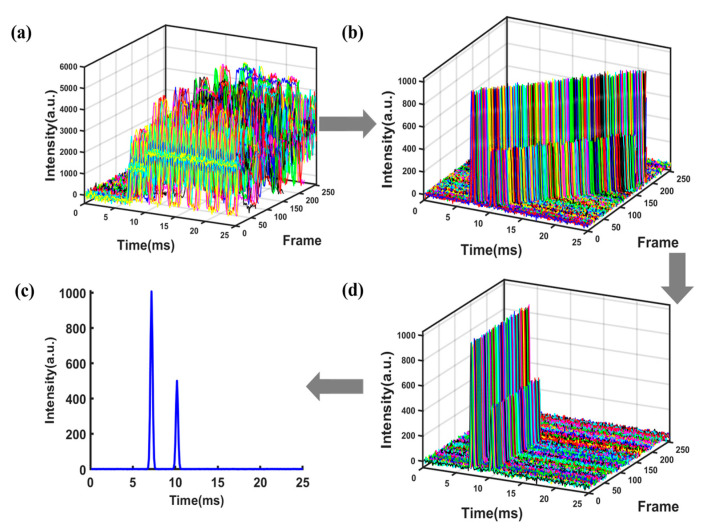
(**a**) The raw data obtained by sampling. (**b**) The group of spectra obtained by deconvolution. (**c**) The spectra obtained by cyclic shift. (**d**) The spectrum obtained by averaging.

When the coefficient matrix W is replaced with $H_{n-1}$, the theoretical gain of SNR is given by $\sqrt{n-1}/2$. The set of spectra $\left(f_{1},f_{2}\ldots f_{i}\ldots f_{n}\right)$ obtained by deconvolution from Equation (6) represents a collection of spectra, where $f_{1}$ corresponds to the desired spectrum. Since each spectrum $f_{i}$ is obtained by introducing a time delay to the previous spectrum $f_{i-1}$, the gain of SNR can be further improved by cyclic shift averaging. The signal modulation and processing flowcharts and simulations are shown in Figure 4 and Figure 5 (for clarity, different frames are marked with different colors), respectively. 

**Figure 6:**
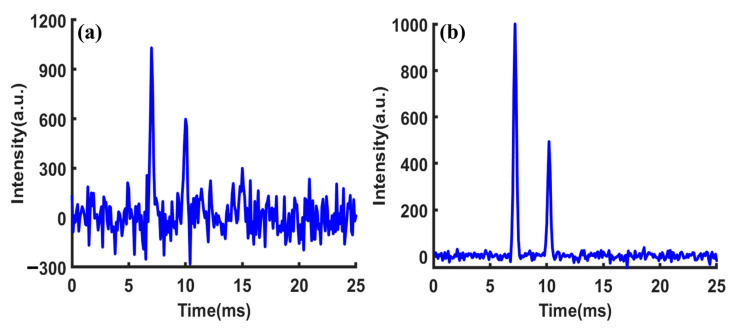
Simulated spectrum of (**a**) single pulse; (**b**) the first spectrum of the group of spectra obtained by deconvolution.

The SNR of the single-pulse IMS in Figure 6a was 10.1. Figure 6b shows the first spectrum obtained through the inverse transform of Sylvester-HT-IMS, with a SNR of 81.5. The gain of SNR relative to the single-pulse IMS was 8.06, which is very close to the theoretical value of 7.98. This demonstrates the correctness of the proposed theory.

## 3. Experiment

### 3.1. Instruments

**Figure 7:**
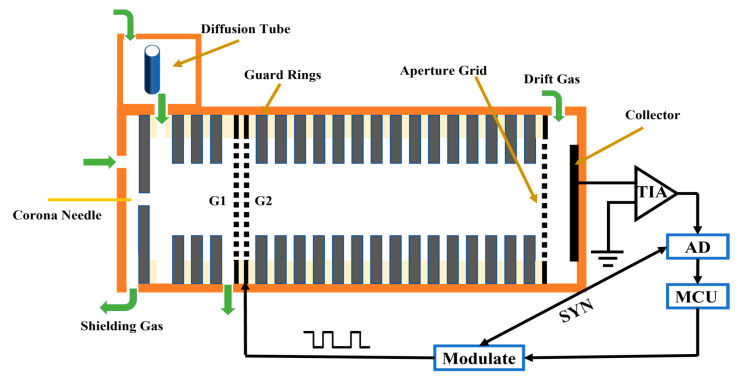
The structural diagram of IMS for experiment.

A home-made IMS with corona discharge ionization source constructed at Soochow University was used for the experiment and its detailed structure diagram is shown in Figure 7. The spectrometer comprised five primary components: sampler, ionization source, ion gate, drift tube, and data acquisition system. A geometry of point-to-ring was installed at the top of the IMS cell to generate reactant ions through direct current (DC) corona discharge. The corona needle and the opposite electrode were positioned at a distance of 2 mm, with the corona voltage set to 6000 V and the opposite electrode at 3000 V. In this experiment, the TPG (Tyndall and Power gate) was used as the ion gate, with a 0.5 mm spacing between G1 and G2, and a reverse electric field of 95 V/mm. The drift tube had a length of 50 mm and consisted of alternating metal ring electrodes (1.35 mm thickness) and polytetrafluoroethylene rings (1.15 mm thickness). Each metal ring electrode was connected by a series of 2 MΩ resistors, forming a uniform electric field of 35 V/mm along the central axis of the drift tube. The carrier gas and the drift gas used were clean air filtered by activated carbon and 13 × molecular sieve, with a flow rate of 100 mL/min and 200 mL/min, respectively. The corona discharge ionization source utilized a protection gas with a flow rate of 700 mL/min. In the experiment, the current generated by the Faraday disk was converted into a voltage signal using a homemade trans-resistance amplifier with a bandwidth of 8 kHz and a gain of 1.2 GV/A. The temperature of the IMS tube was controlled at 70 °C. Two commercially high voltage power supply modules were purchased from Dongwen High Voltage Power Supply Co., Ltd. (Tianjin, China) for the DC corona discharge and the drift tube.

### 3.2. Data Acquisition and Processing

The signal acquisition for SA-IMS, HT-IMS and Sylvester-HT-IMS was performed using a data acquisition card (model USB6210, National Instruments, Austin, TX, USA) based on LabVIEW (National Instruments, Austin, TX, USA). The sampling rate of SA-IMS was 40 kHz and the sampling rate of HT-IMS and Sylvester-HT-IMS was 10 kHz. The modulation code for Sylvester-HT-IMS and HT-IMS was generated by a homemade board-level circuit equipped with a programmable microcontroller unit (model STM32F429, STMicroelectronics, Geneva, Switzerland). The modulation timing was precisely synchronized with the data acquisition card’s sampling process through the internal trigger signal of the data acquisition card. The signals collected from the three kinds of IMS were subsequently transmitted to MATLAB for analysis.

### 3.3. Reagents

Standard chromatography-grade pure sample solutions of methyl salicylate (MESA) was purchased from Shanghai Macklin Biochemical Co., Ltd. (Shanghai, China). To maintain a constant concentration of the sample gas, a trace gas generator purchased from Suzhou Weimu Intelligent System Co., Ltd. (Suzhou, China) was used in the experiment. Initially, 3 mL MESA was placed into homemade diffusion tubes, and the ends were tightly sealed with Polytetrafluoroethylene (PTFE) plugs. The diffusion tube and plugs were then secured using stainless steel metal sleeves. The sealing of the diffusion tube was tested to ensure no sample solution leakage. The carrier gas introduced into the trace gas generator to dilute the gas diffusing out of the diffusion tube was generated by the micro air pump with the model D50H-42H, which was purchased from Chengdu Hailin Technology Co., Ltd. (Chengdu, China). The flow rate of the diluted gas could be adjusted from 0 to 17 L/min. The gas diffused from the diffusion tube combined with the carrier gas, purified by a molecular sieve which was purchased from Sinopharm Chemical Reagent Co., Ltd. (Shanghai, China) to form a mixed sample gas.

## 4. Discussion

### 4.1. The Measurement of Reactant Ions Using SA-IMS, HT-IMS and Sylvester-HT-IMS

**Figure 8:**
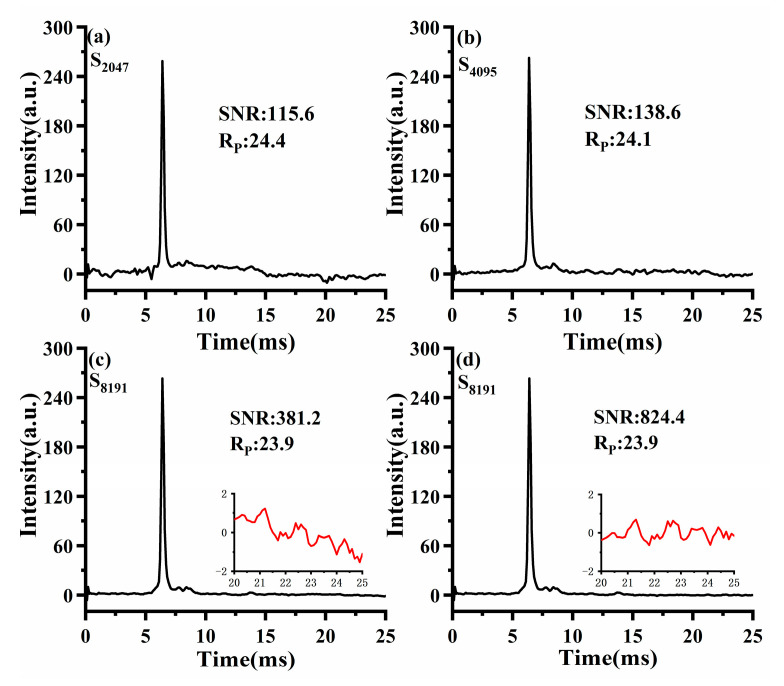
The spectra of HT-IMS under PRBS with different length: (**a**) 2047; (**b**) 4095; (**c**) 8191; (**d**) the corrected spectrum.

To facilitate a fair comparison between Sylvester-HT-IMS and HT-IMS, the signal acquisition time remained consistent for HT-IMS and Sylvester-HT-IMS modulated by a 255-order matrix. Figure 8a–d display the spectra of HT-IMS for reactant ions obtained by modulating by PRBS of varying lengths at a pulse width of 100 μs. As with the conventional approach, the ratio of an ion’s drift time to half of the peak width is used to indicate the size of Rp. The calculation rule of SNR is the relative height between the ion peak and baseline divided by the standard deviation of the baseline of the spectrum between 20~25 ms. Specifically, using the execution time of $H_{255}$ Sylvester-HT-IMS as the benchmark, in total about 9.05 s, $S_{8191}$ HT-IMS will average 11 times, $S_{4095}$ HT-IMS will average 22 times, and so on. It can be observed that the SNR of HT-IMS increases with the length of the PRBS. However, when the PRBS length is 2047 and 4095, the baseline becomes distorted and there are many spikes on the baseline, which will worsen the SNR of HT-IMS. When the sequence length is 8191, the spikes on the HT-IMS baseline have almost disappeared. However, as is shown in Figure 8c, the baseline of $S_{8191}$ HT-IMS was amplified from 20 ms to 25 ms and marked with a red curve and an obvious distortion of the baseline can also be observed. Although the noise level of $S_{8191}$ HT-IMS is very weak, this distortion of the baseline will seriously deteriorate the SNR of HT-IMS. The fourth-order polynomial is used to fit the fluctuation trend of the noise baseline and subtract the fitting curve, and the SNR of HT-IMS is nearly 2.16 times that before correction, as is shown in Figure 8d. The result of baseline correction indicates that the distortion of the baseline will greatly weaken the SNR of HT-IMS and fail to reflect the capability of noise reduction of HT-IMS itself. In the subsequent test of the product ion, the calculations of SNR for the spectrum of HT-IMS were all performed with baseline correction, but the spectra themselves remained in their original form to better display the differences from Sylvester-HT-IMS. For HT-IMS, a longer sequence implies a larger inverse transformation matrix that needs to be stored. Taking into account the trade-off between noise reduction performance and the cost of storage and computation, in the subsequent experiments, HT-IMS uses a sequence length of 8191.

**Figure 9:**
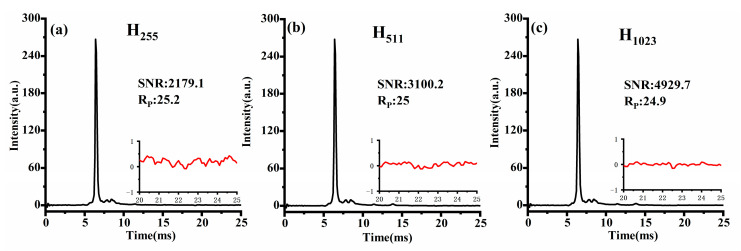
The spectra of Sylvester-HT-IMS under different order matrix modulations: (**a**) 255; (**b**) 511; (**c**) 1023.

Similarly, Figure 9a–c depict Sylvester-HT-IMS for reactant ions modulated by H-matrix with varying orders at pulse width of 100 μs. It is evident that as the matrix order increases, the peak-to-peak value of baseline noise in Sylvester-HT-IMS decreases. Additionally, the intensity of the ion peak is also higher than that of HT-IMS. Considering that the proportion of “1” in the modulation encoding of HT-IMS and Sylvester-HT-IMS is the same, the ion flux theoretically should be consistent. Therefore, the enhancement in the intensity of ion peaks can be attributed to the mathematical description of Sylvester-HT-IMS being more consistent with the actual measurement process of the transfer spectrum, making it a superior measurement method. Overall, the SNR of Sylvester-HT-IMS for reactant ion testing is 5.7 times higher than that of $S_{8191}$ HT-IMS before baseline correction, and the spikes present in HT-IMS are eliminated effectively. At the same time, the Rp of Sylvester-HT-IMS has also been improved compared to that of HT-IMS.

**Figure 10:**
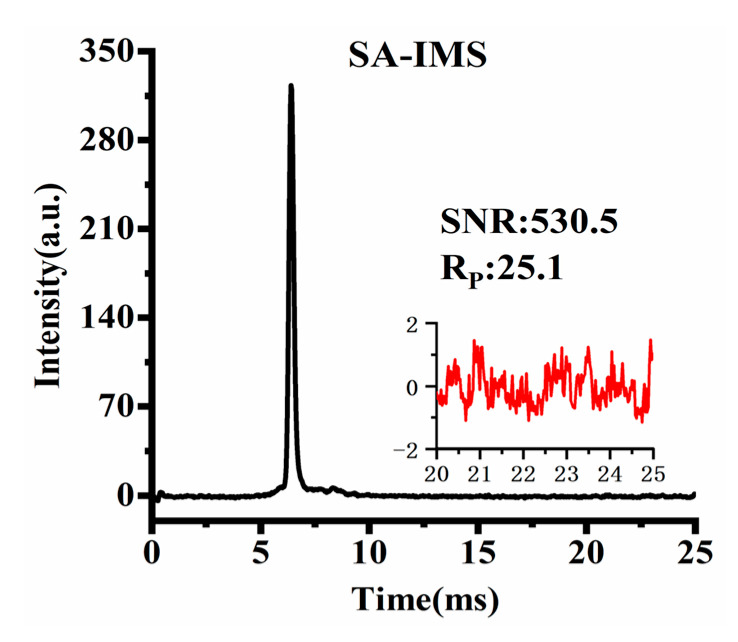
Spectrum of SA-IMS after an average of 355 spectra.

Figure 10 shows the SA-IMS spectrum after an average of 355 spectra, with a total time of 9.05 s, which is consistent with the time required for a single execution of $H_{255}$ Sylvester-HT-IMS. Considering the ion discrimination of the TP ion gate, in order to maintain almost the same Rp as much as possible in the three kinds of IMS, the pulse width of SA-IMS was set to 170μs. At the same signal acquisition time, it can be observed that the SNR of $H_{255}$ Sylvester-HT-IMS is 4.1 times higher than that of SA-IMS, while $S_{8191}$ HT-IMS has a lower SNR than SA-IMS before baseline correction and a higher SNR than $S_{8191}$ HT-IMS after baseline correction. From the comparison of the spectrum obtained by reactant ions, the Sylvester-HT-IMS exhibits a significant advantage on SNR compared to that of HT-IMS and SA-IMS. To make the signal acquisition time and the computational complexity of the deconvolution operation acceptable for the fast detection of IMS, in the subsequent comparative experiments involving three kinds of IMS, Sylvester-HT-IMS will utilize a 255-order H-matrix for modulation and HT-IMS will be modulated by PRBS with a length of 8191. To maintain consistent signal acquisition time, in total about 9.05 s, $S_{8191}$ HT-IMS will be averaged 11 times and SA-IMS will be averaged 355 times.

### 4.2. The Measurement of Product Ions Using SA-IMS, HT-IMS and Sylvester-HT-IMS

**Figure 11:**
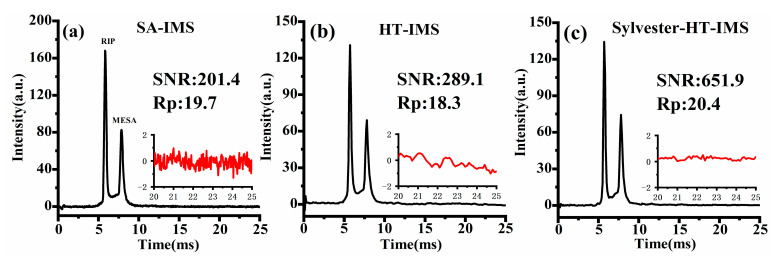
The spectra of MESA: (**a**) SA-IMS; (**b**) HT-IMS; (**c**) Sylvester-HT-IMS.

Furthermore, in order to comprehensively evaluate the performance of SA-IMS, Sylvester-HT-IMS and HT-IMS, product ions produced by MESA were selected. The carrier gas flow rate of 4 L/min was passed into the trace gas generator to dilute the gas sample volatilized from the diffusion tube, and then 100 mL/min gas containing the sample was injected into the IMS cell. SA-IMS, $S_{8191}$ HT-IMS and $H_{255}$ Sylvester-HT-IMS were, respectively, tested under the same signal acquisition time of 9.05 s. The spectral comparison is shown in Figure 11.

As shown in Figure 11a–c, when the MESA sample is introduced into the IMS cell, two distinct peaks are observed in the spectrum. The SNR and Rp of the product ions of MESA are marked. It is evident that the baseline noise level of SA-IMS is significantly higher than that of both HT-IMS and Sylvester-HT-IMS. Despite SA-IMS exhibiting higher ion peak intensity due to larger pulse width, a larger baseline noise will reduce the SNR, which is only 201.4, lower than the 289.1 of the corrected HT-IMS and 651.9 of Sylvester-HT-IMS. It is noteworthy that although both Sylvester-HT-IMS and HT-IMS have a pulse width of 100 μs, the Rp of Sylvester-HT-IMS is approximately 11.4% higher than that of HT-IMS. This may be attributed to the uneven distribution of gate opening in the PRBS used in HT-IMS, which can slightly broaden the peaks in the spectrum due to potential space charge effects between ion clusters. Additionally, the longer number of consecutive 1 s in the PRBS of $S_{8191}$ HT-IMS leads to an extended opening time of the ion gates, increasing the charge number of ion clusters and potentially exacerbating ion diffusion effects during the drift process. The ion peaks in the spectrum of Sylvester-HT-IMS exhibit stronger intensity and lower baseline noise than HT-IMS. Therefore, with the improvement of Rp, the SNR of Sylvester-HT-IMS is 2.26 times as high as that of HT-IMS and 3.23 times as high as that of SA-IMS. The peak area of an ion peak generally represents the corresponding number of detected ions. The ratio of the peak area of the product ions to the reactant ions in SA-IMS, HT-IMS and Sylvester-HT-IMS are 0.72, 0.81 and 0.97, respectively. From the results of this experiment, it can be observed that the number of product ions detected by SA-IMS is significantly smaller than that of reactant ions, indicating a distinct ion discrimination. However, attribute to the characteristic of high throughput, both HT-IMS and Sylvester-HT-IMS show a better performance over SA-IMS on eliminating ion discrimination and the number of different kinds of ions detected in the Sylvester-HT-IMS spectrum is almost the same.

## 5. Conclusions

In this work, the conventional HT-IMS method was carefully examined from its underlying mathematical principle and actual data collection process. Through theoretical analysis and simulation, the distortion of the deconvoluted spectrum was considered to result from the inconsistency between the mathematical model of the original Hadamard transform and the actual data collection process. Therefore, a new mathematical framework was developed according to the IMS data collection, which employs the modulation of the ion gate with a Sylvester-type Hadamard matrix and cyclic shift averaging of deconvoluted data.

From the results of both the simulation and experimental measurement, this novel method, Sylvester-HT-IMS, demonstrated better performance over conventional SA-IMS and HT-IMS, including eliminated baseline distortion, improved SNR and improved ion discrimination.

## Data Availability

The data presented in this work are available in the article.
